# Museum genomics approach to study the taxonomy and evolution of Woolly-necked storks using historic specimens

**DOI:** 10.1093/g3journal/jkae081

**Published:** 2024-04-16

**Authors:** Prashant Ghimire, Catalina Palacios, Jeremiah Trimble, Sangeet Lamichhaney

**Affiliations:** Department of Biological Sciences, Kent State University, Kent, OH 44240, USA; Department of Biological Sciences, Kent State University, Kent, OH 44240, USA; Museum of Comparative Zoology, Harvard University, Cambridge, MA 02138, USA; Department of Biological Sciences, Kent State University, Kent, OH 44240, USA; School of Biomedical Sciences, Kent State University, Kent, OH 44240, USA

**Keywords:** conservation genomics, museum, phylogeny, Woolly-necked storks

## Abstract

The accessibility of genomic tools in evolutionary biology has allowed for a thorough exploration of various evolutionary processes associated with adaptation and speciation. However, genomic studies in natural systems present numerous challenges, reflecting the inherent complexities of studying organisms in their native habitats. The utilization of museum specimens for genomics research has received increased attention in recent times, facilitated by advancements in ancient DNA techniques. In this study, we have utilized a museum genomics approach to analyze historic specimens of Woolly-necked storks (*Ciconia* spp.) and examine their genetic composition and taxonomic status and explore the evolutionary and adaptive trajectories of populations over the years. The Woolly-necked storks are distributed in Asia and Africa with a taxonomic classification that has been a matter of ambiguity. Asian and African Woollynecks were recently recognized as different species based on their morphological differences; however, their genomic validation was lacking. In this study, we have used ∼70-year-old museum samples for whole-genome population-scale sequencing. Our study has revealed that Asian and African Woollynecks are genetically distinct, consistent with the current taxonomic classification based on morphological features. However, we also found a high genetic divergence between the Asian subspecies *Ciconia episcopus neglecta* and *Ciconia episcopus episcopus*, suggesting this classification requires a detailed examination to explore processes of ongoing speciation. Because taxonomic classification directly impacts conservation efforts, and there is evidence of declining populations of Asian Woollynecks in Southeast Asia, our results highlight that population-scale studies are urgent to determine the genetic, ecological, and phylogenetic diversity of these birds.

## Introduction

Population divergence and speciation are still central subjects in evolutionary biology (Engelbrecht *et al*. 2020; Liu *et al*. 2020), and modern research in evolutionary biology has provided new insights into the mechanisms underlying population divergence and speciation (Bolnick *et al*. 2023). Advances in genomics and molecular biology have allowed scientists to examine the genetic basis of speciation and identify genes involved in reproductive isolation and divergence (Coyne 1992; Schluter 2009; Sobel *et al*. 2010). Additionally, studies using model organisms and comparative genomics have shed light on the different modes and rates of speciation (Gagnaire 2020; Genereux *et al*. 2020). With the availability of large-scale genomics data, characterization of the dynamic interplay of diverse evolutionary processes such as gene flow, mutation, recombination, drift, and selection in shaping the genomic landscape of speciation has been increasingly feasible (Ravinet *et al*. 2017; Campbell *et al*. 2018).

However, conducting genomic studies in nonmodel natural populations comes with several challenges, reflecting the complexities inherent in studying organisms within their native environments. Many such species reside in remote or difficult-to-access locations, posing logistical challenges for researchers in terms of transportation, equipment, and personnel. Natural history collections that house a vast wealth of specimens that are systematically cataloged can serve as a viable alternative for genomic studies on these natural populations and overcome sampling limitations associated with fieldwork, especially for species that are elusive, rare, or difficult to study in their natural habitats (Krell and Wheeler 2014; Meineke *et al*. 2019). The use of museum specimens for genomics research has gained popularity in recent years as advancements in high-throughput genome sequencing technologies have made it possible to obtain valuable genomic data even from low-quality or historical biological samples (Card *et al*. 2021). Museum genomics—the application of genomics research using traditional museum and cryogenic collections—has significantly elevated scientific research, particularly in the fields of ecology and evolutionary biology (Raxworthy and Smith 2021).

The genetic and phenotypic differences among populations are commonly shaped by the environment (Bonaccorso *et al*. 2006; Hernández-Romero *et al*. 2018). Geography plays a major role in separating well-connected populations reducing gene flow in favor of divergence and reproductive isolation (Coyne 1992). Related taxa spanning continents undergo disparate influences, including dynamic landscape alterations, climatic variations, seasonal shifts, and ecological interactions. Studies have indicated that the divergence time of sister clades located on different continents aligns with continental break-ups, providing additional evidence supporting the role of plate tectonics and its impact on biodiversity (Carney and Dick 2000; McIntyre *et al*. 2017). The study of closely related species distributed across continents serves as a valuable approach for characterizing the evolutionary trajectories of populations. It allows us to discern the impacts of environmental factors and climate change, providing insights into how geographical events have shaped the current biodiversity. A key aspect of these studies involves understanding the mechanisms, timing, and contributing factors that led to divergence, ultimately resulting in the formation of distinct taxa, essentially, the process of speciation.

**Fig. 1.**
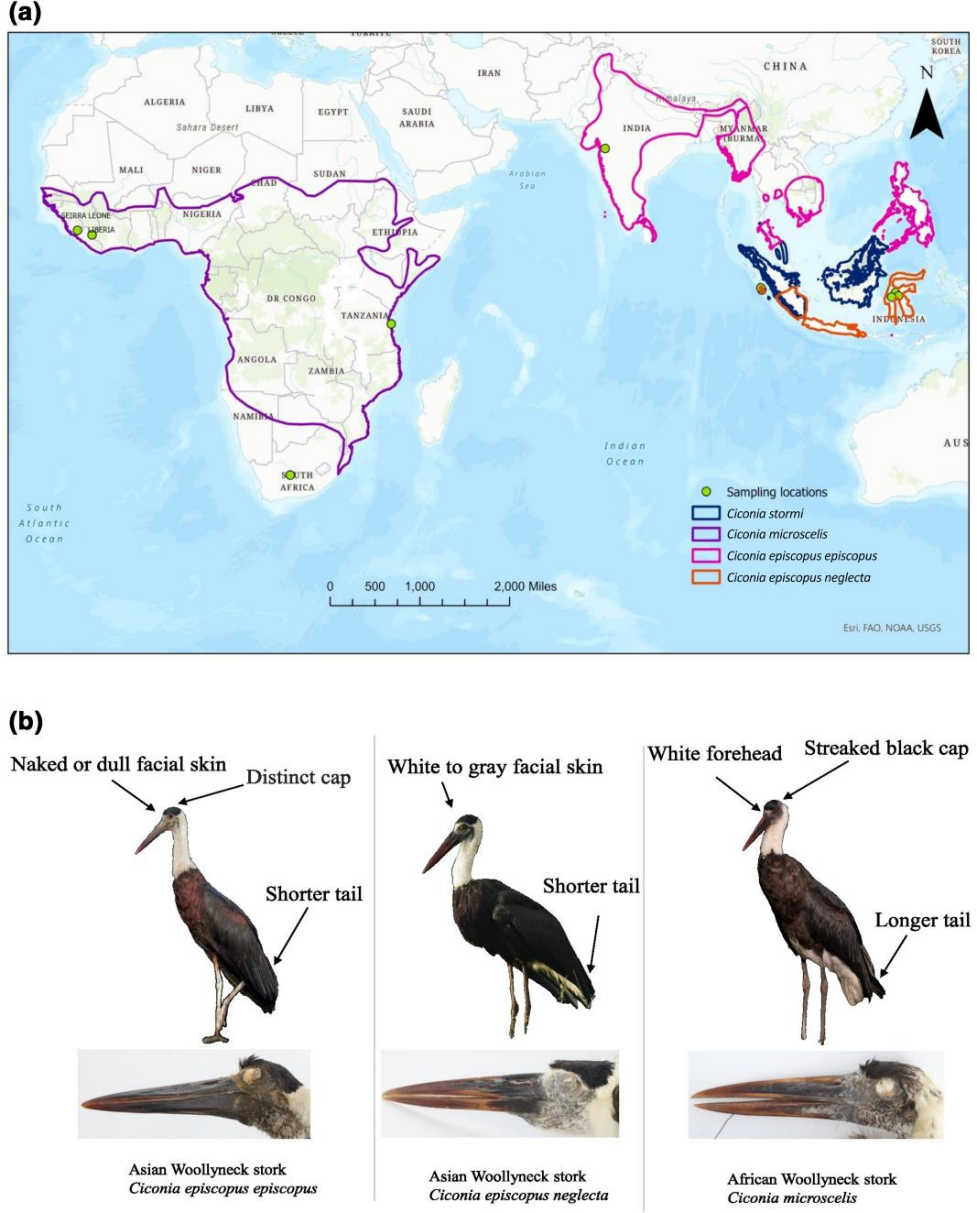
Distribution, sample location, and morphology of storks used in this study: a) Distribution of African and Asian Woollynecks and Storm's storks. Species and subspecies distribution polygons were made using publicly available data from BirdLife International and Handbook of the Birds of the World (2022). The distribution of *C. e. neglecta* is based on data from Mlodinow *et al*. (2022). b) Morphological differences between Woolly-necked storks. Photographs © Vijayandra Desai, Leonardus Adi Saktyari (ML425347741), and Jonah Gula (left to right, bird photos) and Jeremiah Trimble, MCZ, Harvard University (headshot of museum specimens).

One such example of intercontinental divergence and speciation is the Woolly-necked storks (collective term for Asian Woollynecks and African Woollynecks), which are distributed across Africa including sub-Saharan Africa and Asia including the southeastern islands (Birdlife International 2023) (Fig. 1a). African and Asian Woollynecks were previously regarded as a single species until 2010. However, due to observed differences in their morphology and plumage coloration, 2 separate species have been officially recognized: the Asian Woollynecks (*Ciconia episcopus*) and the African Woollynecks (*Ciconia microscelis*) (Tobias *et al*. 2010; del Hoyo and Collar 2014). They are large wading birds, measuring ∼86–95 cm in height, with a predominantly black body and a white neck that appears fluffy due to elongated feathers (Birdlife International 2023). Asian Woollynecks have a characteristic distinct black cap with naked or dull facial skin and a shorter tail, while African Woollynecks have a streaked black cap with white facial skin and a longer tail (Mlodinow *et al*. 2022) (Fig. 1b). Although Asian and African Woollynecks are primarily found in wetland and farmland habitats, there is a growing observation of their presence in urban environments (Thabethe and Downs 2018; Hasan and Ghimire 2020). The urban association is particularly pronounced in the African Woollynecks, who venture into residential areas and feed on human-provided food (Thabethe and Downs 2018).

The reclassification of Woolly-necked storks into 2 distinct species has had a significant impact on their conservation status; the status of Asian Woollynecks shifted from “Least Concern” to “Vulnerable” in 2014 (Birdlife International 2020). Presently, it is considered “Near Threatened” based on recent population estimates (Kittur and Sundar 2020). In contrast, the status of African Woollynecks has remained unchanged, continuing to be designated as “Least Concern” (Birdlife International 2020). African Woollynecks appear morphologically homogeneous, and their population size is generally considered to be stable throughout the distribution range (Birdlife International 2020). Contrastingly, Asian Woollynecks are categorized into 2 subspecies due to discernible morphological differences: *C. episcopus episcopus*, located in South Asia, the Maley Peninsula, and mainland Southeast Asia, and *C. episcopus neglecta*, found in Southern Sumatra, Java, and Wallacea of Indonesia (Fig. 1a) (Gill *et al*. 2022; Mlodinow *et al*. 2022). Both subspecies differ in the color of their facial skin and iris (del Hoyo *et al*. 2020). The demographic status of Asian Woollynecks varies significantly across their distribution range. Populations in India, Sri Lanka, and Nepal have shown relative stability or even an increase (Ghimire *et al*. 2021) compared with Southeast Asia such as the Philippines, where these birds have faced localized extinctions, potentially linked to habitat deterioration, wetland degradation, pollution, and increased hunting activities (Birdlife International 2020; Kittur and Sundar 2020; Ghimire *et al*. 2021).

The taxonomic classification of *C. e. neglecta* and *C. e. episcopus* as subspecies has been a longstanding subject of debate, primarily stemming from unclear morphological descriptions and a lack of knowledge about the distribution of these subspecies. Early accounts of this bird highlighted morphological differences, particularly the red coloration on the bill, between the mainland South Asian and Indonesian island populations (Riley 1924). This led to the proposal of *C. e. neglecta* being a subspecies (Finsch 1904), a classification that was debated due to insufficient evidence and the presence of birds exhibiting both morphologies in the Malay Peninsula (Hancock *et al*. 1992; Holmes 1996). Despite the identification of additional morphological differences (Fig. 1b), the ongoing debate is fueled by a scarcity of reliable information regarding the distribution and ecology of these subspecies (Mlodinow *et al*. 2022). Questions persist regarding the existence of contact or isolation between each subspecies, particularly in Southeast Asia, further adding to the uncertainties. This lack of clarity holds potential implications for the conservation of these species (Sundar 2020) and may significantly impact conservation efforts aimed at their populations (Gutiérrez and Helgen 2013; Zachos 2013). Genomic studies of these species have the potential not only to examine their current taxonomic status and unravel their evolutionary history but also to provide valuable resources for a comprehensive exploration of the molecular processes underlying their adaptation to local environments across their extensive distribution range.

In this study, we employed a whole-genome sequencing approach using museum specimens to investigate the genetic diversity of Woolly-necked storks and examine their taxonomy and evolutionary history. Our findings contribute to the genomic validation of their current taxonomic status and explore its potential implications for conservation efforts and population management. This study provides a roadmap on how the museum genomics approach can be utilized to study centuries-old specimens to gain insights into the genetic composition, confirm their taxonomic status, and explore how populations have evolved and adapted over the years.

## Materials and methods

### Obtaining museum specimens

We conducted a search query using the keyword “Ciconia” in the VertNet online database—a platform for sharing and accessing biodiversity data from diverse biological collections worldwide (http://vertnet.org/). Our search identified specimens of both Asian and African Woollynecks were housed at the Museum of Comparative Zoology (MCZ) at Harvard University. From the MCZ, we obtained toe-pad skin samples from a total of 13 museum specimens, including 6 from Asian Woollynecks (*C. episcopus*), encompassing both subspecies (3 *C. e. episcopus* and 3 *C. e. neglecta*), 5 from African Woollynecks (*C. microscelis*), and 2 from Stormi’s stork (*Ciconia stormi*), which we utilized as an outgroup (Mlodinow *et al*. 2022). These specimens were collected from various locations in Asia and Africa between 1934 and 1953 (Supplementary Table 1).

### DNA extraction and whole-genome sequencing

DNA extraction from the toe-pad skin samples was performed using the DNeasy Blood and Tissue Kit (Qiagen, Valencia, CA, United States) with a modified version of the protocol suggested by Harvey *et al*. (2020). Given that the samples were over 70 years old, we adhered to the recommended guidelines for ancient DNA analysis to mitigate the risk of environmental contamination (Orlando *et al*. 2021). To prevent cross-contamination, all laboratory procedures were conducted in a biosafety cabinet dedicated exclusively to ancient DNA analysis, where no fresh samples were processed. Additionally, all equipment used in the extraction process was carefully cleaned and sterilized before use.

The quantification of extracted DNA was performed using a Qubit fluorometer (Thermo-Fisher, Waltham, MA, United States). However, 1 sample from the Asian Woollyneck yielded an insufficient amount of DNA and was consequently excluded from subsequent procedures. The DNA from the remaining 12 samples underwent library preparation and whole-genome sequencing, carried out by a commercial genome sequencing provider Admera Health, New Jersey, United States. Illumina library preparation kit was used to generate genome sequencing libraries with an average fragment size of ∼400 bp. The sequencing libraries were multiplexed and sequenced in a single lane of S4 flow cell in the Illumina NovaSeq 6000 platform, generating 150 bp paired-end reads. The sequencing process aimed to achieve ∼10× coverage for each sample. The number of reads obtained per sample is in Supplementary Table 2.

### Sequence alignment and variant calling

All obtained reads were quality-checked using FASTQC (https://www.bioinformatics.babraham.ac.uk/projects/fastqc/). We then mapped the reads from each individual against the reference genome of Maguari stork (*Ciconia maguari*) (BioProject PRJNA715733) (Feng *et al*. 2020) using BWA v0.7.17 with default parameters (Li and Durbin 2009). The Maguari stork genome was the closest relative of Woolly-necked storks available in public databases at the time of the analysis. We checked the alignments for PCR duplicates using PICARD (http://picard.sourceforge.net/) and used the Genome Analysis Toolkit (GATK) v4.2.0.0 (McKenna *et al*. 2010) for calling genetic variants using default parameters. We used GATK best practice recommendations (Van der Auwera *et al*. 2013) of base quality recalibrations (minimum median base quality > 20) and insertion/deletion (INDEL) realignment before calling variants. Raw variants were filtered for quality downstream using an in-house pipeline previously developed in the lab (Lamichhaney *et al*. 2015) by only selection SNPs with parameters, Fisher strand bias > 100.0, mapping quality < 50.0, mapping quality rank sum < −5.0, read position rank sum < −4.0, base quality rank sum < −5.0, depth > 500 and < 10, quality by depth score < 5.0, SNP quality > 100, and genotype quality > 10 (see details on each parameter here: https://gatk.broadinstitute.org/hc/en-us/articles/360035531692-VCF-Variant-Call-Format). We further calculated the percentage of missing SNPs across samples using VCFTools v0.1.13 (Danecek *et al*. 2011) and kept only the SNPs present in all 12 samples for downstream analysis.

### Phylogeny reconstruction and estimates of genetic diversity

We utilized the SNP data generated above to produce an approximate maximum-likelihood phylogeny with FastTree v0.2.1 using recommended default parameters for nucleotide alignments (Price *et al*. 2010). To assess the local support for each node in the phylogenetic tree, we employed the Shimodaira–Hasegawa test implemented in FastTree. In addition to using methods to generate a single “species” tree by concatenating all possible “gene” trees, we also used SNAPP (Bryant *et al*. 2012), which implements a full coalescent model to examine all possible gene trees across the genome.

We further assessed genetic diversity within and between species using VCFtools v0.1.13 (Danecek *et al*. 2011) by computing genetic metrics such as nucleotide diversity (π), heterozygosity, inbreeding coefficient (*F*), and fixation index (*F*_ST_). For the calculation of nucleotide diversity and inbreeding coefficient (*F*), we made separate VCF files with SNPs present in at least 1 sample within that population.

### Divergence time estimates

To infer a time-calibrated tree, we used SNAPP (Bryant *et al*. 2012). The genus *Ciconia* diverged from its sister taxa 8.78 MYA, based on TimeTree database estimates (http://www.timetree.org/); however, there were no divergence time records of any of the clades associated with species being used in this study. We, therefore, used divergence time estimates between White stork *Ciconia ciconia* and Oriental stork *Ciconia boyciana* to constrain the relative root age with 0 offsets and a standard deviation of 0.005 MY between Stormi's stork *C. stormi* and Woolly-necked stork *C. episcopus*. *C. ciconia* and *C. boyciana* are sister clades that have diverged recently and demonstrate subtle morphological differences similar to *C. episcopus* and *C. stormi* (Hancock *et al*. 1992; Slikas 1997; de Sousa *et al*. 2023); hence, we used their estimates as a proxy for *C. episcopus*/*C. stormi* divergence time estimates.

For divergence time estimates using Bayesian inference, we ran BEAST v.2.6.3 (Bouckaert *et al*. 2019) for 5 million generations with sampling conducted every 250 generations; the initial 10% was discarded as burn-in. To ensure convergence of all parameters (ESS > 200), Tracer 1.7.2 (Rambaut *et al*. 2018) was employed, and a maximum clade credibility tree was generated using TreeAnnotator 2.6.4 (Drummond and Rambaut 2007).

### Population structure and ancestry

We examined the genetic differentiation among samples using a principal component analysis (PCA) with PLINK v.1.9 (Chang *et al*. 2015). As 1 major assumption of a PCA is independent data, we first pruned our SNP data set considering linkage disequilibrium (LD). We removed any SNP that shows an *r*^2^ > 0.1 within a 50 kb window and step size of 10 bp and kept a total of 1,780 SNPs for the PCA. We plotted the PCA output in R (Team 2016).

We further estimate the ancestry of individuals based on a genome-wide SNPs data set using Admixture v.1.3.0 (Alexander *et al*. 2009) which utilizes a maximum-likelihood approach. To determine the optimal number of genetically distinct clusters (*K*) that best represent our data, we conducted an exploratory analysis using a cross-validation procedure. This analysis was performed with the *K*-means method implemented in Admixture. We generated a plot to visualize the proportion of ancestry attributed to each cluster and sample using R (Team 2016).

### Examine the evidence of gene flow between species

We utilized ABBA-BABA tests, a statistical method used to detect and quantify gene flow between populations by examining patterns of allele sharing at specific genomic loci using Dsuite (Malinsky *et al*. 2021). The test relies on the principle of the coalescent theory, which describes the stochastic process by which gene lineages trace back to a common ancestor (Green *et al*. 2010; Durand *et al*. 2011). We examined the possible evidence of gene flow between Asian and African Woollynecks (Supplementary Table 6). We chose 4 populations: P1/P2, 2 subspecies of Asian Woollynecks representing sister groups; P3, population of African Woollynecks; and P4, Stormi's stork as an outgroup. In a scenario without gene flow, we expect equal occurrences of 2 possible allelic patterns: ABBA and BABA (ABBA, an allele is shared between P2 and P3 but not with P1, and BABA, an allele is shared between P1 and P3 but not with P2). The D-statistic (also known as the ABBA-BABA statistic) is computed as (ABBA − BABA)/(ABBA + BABA). A positive D-statistic suggests gene flow or introgression from population P2 to population P3. Statistical significance is determined by comparing the observed D-statistic to a distribution of D-statistics generated through simulations (Green *et al*. 2010; Durand *et al*. 2011). If the observed D-statistic falls outside the expected range, it indicates a significant deviation from the null hypothesis of no gene flow.

To further examine the evolutionary history and patterns of gene flow in Woollynecks, we employed fastsimcoal2 (Excoffier *et al*. 2021). We first generated site frequency spectrum data based on sequence alignment files using easySFS (Gutenkunst *et al*. 2009). We then tested models for 3 gene flow scenarios among Asian and African Woollynecks: (1) no gene flow, (2) early gene flow, and (3) ongoing gene flow (Supplementary Fig. 7). We carried out 200,000 simulations and 100 expectation-conditional maximization cycles for each model. We repeated the simulation 40 times for each model, which helped us estimate the global maximum likelihood for each model. We used the simulation with the highest estimated likelihood to calculate the Akaike Information Criterion (AIC) value to identify the best-fit model (Meier *et al*. 2017).

## Results

### Quality of DNA from historic museum specimens and their resulting genomic data

The amount and quality of DNA obtained, along with the quality of the resulting genome sequence data, are critical aspects in genomic studies involving historic museum specimens. We obtained sufficient DNA for whole-genome sequencing using toe-pad skin samples from 12 (out of 13) museum specimens (Supplementary Table 2). The amount of whole-genome sequence data generated from these samples was as expected (∼10 Gb of genomic reads per sample, Supplementary Table 2). Various sequence quality parameters such as (1) per base sequence quality, (2) per sequence quality scores, (3) per base sequence content, and (4) per sequence GC content were indicative of good-quality sequence data (Supplementary Fig. 1). However, the fragment size of the genomic DNA extracted from all 12 samples was low, indicating DNA degradation (Supplementary Fig. 2), which is expected for DNA obtained from dry toe-pad skin samples collected more than 70 years ago.

### Alignment of reads to the reference genome of the Maguari stork (*C. maguari*)

The sequence mapping rate ranged from 83.2 to 99.34% across our 12 samples (Supplementary Table 3). One specimen of *C. stormi* (MCZ: 170531) from 1934, the oldest among the samples we collected, had the lowest mapping rate (83.2%). The average genome-wide sequencing depth ranged from 4.5 to 6.9 (Supplementary Table 3). Following our pipeline, we obtained ∼13.5 million good-quality SNPs (Supplementary File 1, see Materials and methods for details). However, as the genomic DNA was degraded for most of the specimens (Supplementary Fig. 2), the rate of missing SNPs across samples was high (Supplementary Table 4). We found the number of missing SNPs in a sample was strongly correlated with the quality of the genomic DNA (Supplementary Fig. 3). Hence, to remove bias due to missing SNPs, we followed a conservative approach and only used 33,087 SNPs that were genotyped in all 12 samples (missingness = 0) for downstream analyses.

### Genome-based phylogeny

**Fig. 2.**
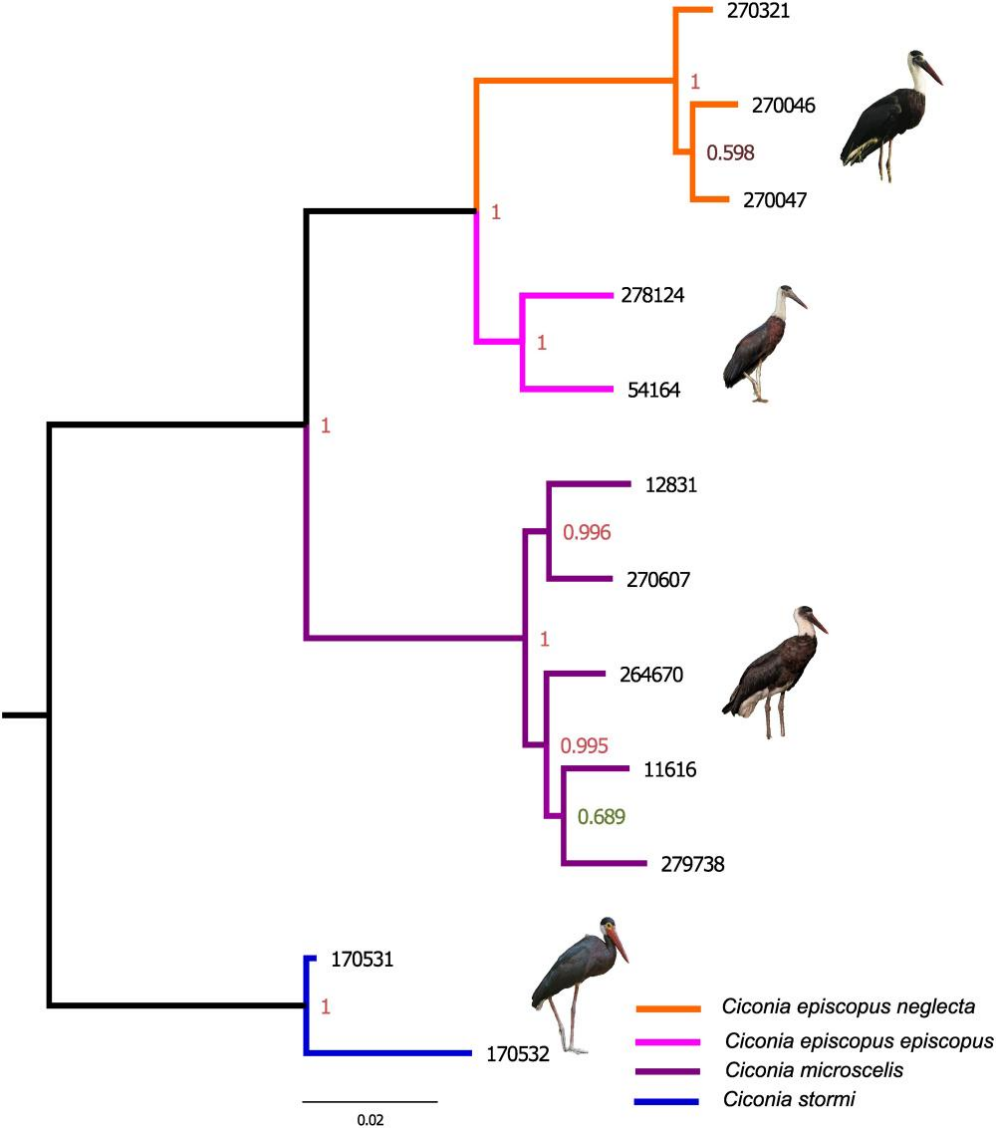
Phylogeny of Woolly-necked storks: maximum-likelihood phylogenetic tree based on 33,087 genome-wide SNPs. Local support values at each node were calculated based on the Shimodaira–Hasegawa test (Price *et al*. 2010). Photographs © Leonardus Adi Saktyari (ML425347741), Vijayandra Desai, Jonah Gula, and James Eaton (ML205762641) (from top to bottom).

We constructed a phylogenetic tree using the filtered data set of 33,087 SNPs, employing a maximum-likelihood approach (Price *et al*. 2010) and using Storm’s stork samples to root the tree as an outgroup. The African and Asian Woollyneck samples formed 2 reciprocally monophyletic clades (Fig. 2). Within the Asian clade, the samples of each subspecies of Asian Woollyneck, *C. e. neglecta*, and *C. e. episcopus* clustered together, demonstrating distinct genetic groups. In the African clade, the branch lengths are shorter, and the node supports are slightly lower than in the Asian clade. African samples also formed 2 distinct clades but unrelated with the geographical distance among them: Clade 1, samples from South Africa, Sierra Leone, and Liberia, and Clade 2, samples from Tanzania clustered together with the sample of unknown origin (Fig. 2, Supplementary Table 1).

We further dated the time of divergence in the phylogenetic tree using Bayesian inference (see Materials and methods for more details). We estimated that the Asian Woollyneck *C. episcopus* and African Woollyneck *C. miscroscelis* diverged 3.34 MYA soon after the divergence of the Woolly-necked stork from Stormi's stork (Supplementary Fig. 4). We also estimated that 2 subspecies of Asian Woollyneck *C. e. episcopus* (from mainland Asia) and *C. e. neglecta* (from Indonesia) diverged 0.96 MYA.

Apart from employing techniques to construct a single “species” tree by concatenating all possible “gene” trees across the genome, we also utilized a method incorporating a comprehensive coalescent model (Bryant *et al*. 2012) to scrutinize all potential gene trees throughout the genome. Our observations revealed that most posterior gene trees exhibited a consistent topology (Supplementary Fig. 5), signifying increased confidence in the proposed phylogenetic relationship among these species/populations being studied.

### Genetic diversity among groups

Average genome-wide nucleotide diversity (pi) was lower in the African population compared with others including the outgroup (Table 1). The inbreeding coefficient (*F*) was close to zero for all samples (excluding the outgroups), indicating a more outbred population with a broader genetic pool. The average genome-wide genetic divergence (*F*_ST_) between Asian and African Woollyneck was estimated as 0.20 (Table 2). The *F*_ST_ between the 2 Asian subspecies was 0.14 whereas between the 2 African clades identified from the phylogenetic tree was −0.006. The *F*_ST_ between *C. e. neglecta* and African samples was higher (0.27) compared with the one between *C. e. episcopus* and African samples (0.16).

**Table 1.** Estimates of genetic diversity.

Group	Average nucleotide diversity (pi)	Average inbreeding coefficient (*F*)
Storm's stork *C. stormi*	1.20 × 10^−4^	0.306
African Woollyneck *C. microscelis*	0.34 × 10^−4^	−0.12
Asian Woollyneck *C. episcopus*	0.81 × 10^−4^	0.15
*C. e. episcopus*	1.84 × 10^−4^	0.106
*C. e. neglecta*	1.55 × 10^−4^	0.24

**Table 2.** Estimates of average genome-wide genetic divergence (*F*_ST_).

Comparison	Average genome-wide *F*_ST_
Asian Woollyneck–Storm's stork (outgroup)	0.25
African Woollyneck–Storm's stork (outgroup)	0.27
Asian Woollyneck–African Woollyneck	0.20
*C. e. episcopus*–*C. e. neglecta*	0.14
African Woollyneck clade 1–African Woollyneck clade 2	−0.006
*C. e. episcopus*–African Woollyneck	0.16
*C. e. neglecta*–African Woollyneck	0.28

**Fig. 3.**
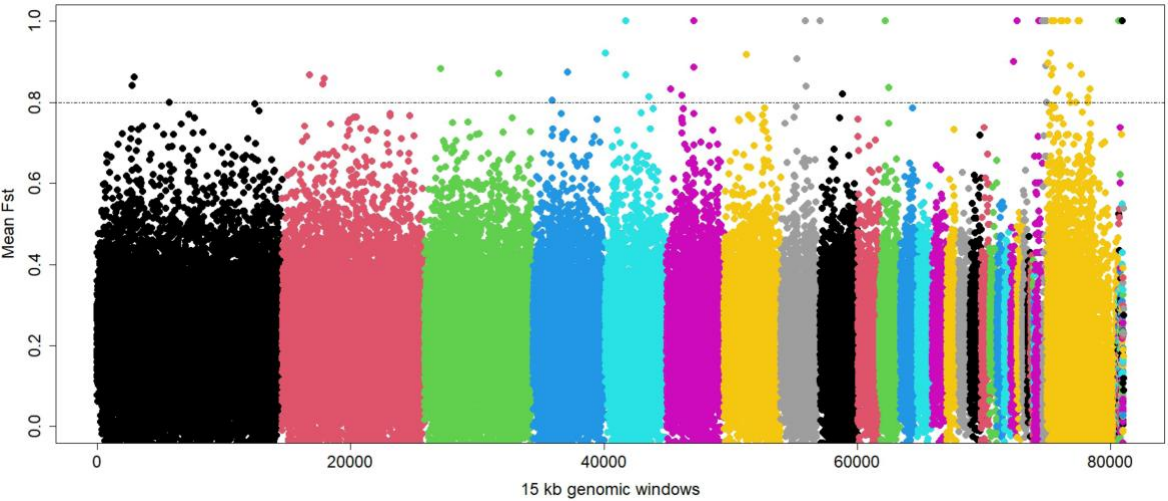
Genomic regions of differentiation between African and African Woollynecks: genome-wide *F*_ST_ calculated for each 15 kb genomic window between African and African Woollynecks. All SNPs from a respective chromosome are coded differently. Genomic regions with high *F*_ST_ (> 0.8) are shown above a dashed horizontal line.

We also calculated *F*_ST_ in 15 kb windows across the genome comparing Asian and African Woollynecks (Fig. 3). Our previous study (Lamichhaney *et al*. 2015) had identified 15 kb as an optimal window size for such *F*_ST_ analysis in bird genomes. We used the top 5% of the genomic windows with *F*_ST_ > 0.8 as an arbitrary cutoff to identify 72 genomic windows showing possible islands of genomic divergence between Asian and African Woollynecks. However, these results are only used to examine the overall landscape of genomic divergence between Asian and African Woollynecks, and we have opted not to delve deeply into outlier SNPs due to the limitations inherent in museum samples and the higher levels of DNA degradation they often exhibit. These samples are not optimal for characterizing outlier SNPs and exploring the functional aspects of divergent genomic regions. Such in-depth analysis is better suited for genome sequence data obtained from fresh, high-quality DNA samples.

### Population structure and ancestry

**Fig. 4.**
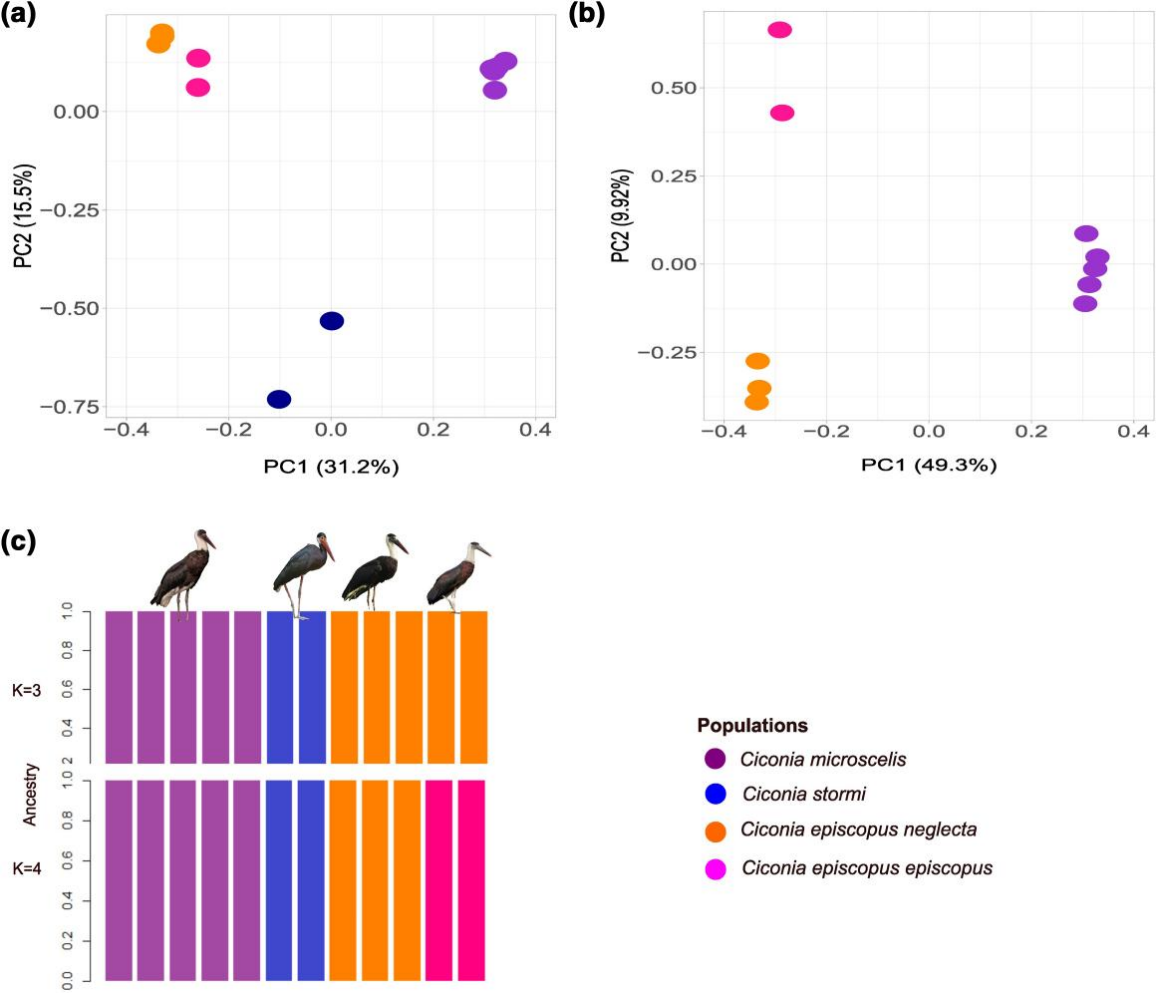
The population structure of Woolly-necked storks was assessed through PCA, both with (a) and without (b) the outgroup Storm's stork. The results reveal distinct clusters for African Woollynecks, Asian Woollynecks, and Storm's stork and differentiate between the Asian subspecies *C.* e*. neglecta* and *C.* e*. episcopus*. c) The admixture plot for *K* = 3 illustrates distinct clusters for samples corresponding to Storm's stork, African Woollynecks, and Asian Woollynecks. Furthermore, for *K* = 4, the plot reveals a finer separation within the Asian Woollyneck, distinguishing between subspecies *C.* e*. neglecta* and *C.* e*. episcopus*. Photographs © Jonah Gula, James Eaton (ML205762641), Leonardus Adi Saktyari (ML425347741), and Vijayandra Desai (from left to right).

PCA indicated first, and second principal components (PCs) explained almost 50% of the genetic variation among the samples (PC1 = 31.2% and PC2 = 15.5%; Supplementary Fig. 6). The first principal component separates the Asian Woollynecks, the African Woollynecks, and Storm's stork groups, while the second principal component mainly separates the outgroup Storm's stork from all Woolly-necked storks (Fig. 4a). Across the first principal component, the Asian samples make 2 distinct genetic clusters corresponding to *C. e. neglecta* and *C. e. episcopus*, while the African samples are not differentiated forming a single cluster. In a PCA excluding the outgroup Storm's stork, the differences between *C. e. neglecta* and *C. e. episcopus* are even more evident, while the African samples still make a single tight cluster (Fig. 4b).

We assessed various values of *K* using a cross-validation procedure and identified that *K* = 3 or 4 is optimal for admixture analysis (Supplementary Table 5). *K* = 3 admixture run grouped samples into Asian Woollynecks, African Woollynecks, and Storm's stork, whereas the *K* = 4 run further divided the Asian samples into the 2 groups, consistent with their subspecies status, *C. e. episcopus* and *C. e. neglecta* (Fig. 4c).

### Gene flow among populations

We examined the possible evidence of gene flow between Asian and African Woollynecks using the ABBA-BABA test (Supplementary Table 6). We did not find evidence of ongoing gene flow between Asian (*C. episcopus*) and African Woollyneck (*C. microscelis*) (*Z* score < 3, *P* > 0.05). We also did not find any incongruent gene trees, and all gene trees match the expected species tree (Supplementary Fig. 5). This further provides a lack of evidence of recent gene flow among different clades across the phylogenetic tree.

To study the evolutionary history and patterns of gene flow between Asian and African Woollynecks, we further utilized fastsimcoal demographic modeling (Excoffier *et al*. 2021) based on the site frequency spectrum. We tested 3 possible scenarios of gene flow patterns (Supplementary Fig. 7). The lowest AIC value supported the best-fit model for the “early gene flow” model.

## Discussion

In this study, we have conducted population genomics analysis on 12 museum specimens collected ∼70 years ago to investigate the evolutionary relationships of Woolly-necked storks, whose taxonomic status has been a topic of uncertainty (Sundar 2020). To our knowledge, this is the first study to produce genomic resources for the Woolly-necked storks and employ the genomic data to examine the existing taxonomy, which has solely relied on morphological data to date. We appreciate the fact that 12 samples are sparse and do not include populations across the entire distribution range. However, it is important to note that this study only focused on using museum samples, which were subject to availability constraints, and obtaining museum samples or freshly collected samples across the entire distribution range was beyond the scope of the current study. However, we have conducted high-coverage whole-genome sequencing, instead of using approaches that use a limited set of genetic markers such as mitochondria, microsatellites, or RADseq; hence, we believe that 12 samples were adequate for this study.

Based on a genome-wide phylogeny, PCA, ancestry analysis, and various metrics of genetic diversity, our results have revealed that Asian and African Woollynecks are genetically distinct sister clades, lending support for the current taxonomy of these birds which recognizes Asian and African Woollynecks as distinct species, *C. episcopus* and *C. microscelis* (del Hoyo *et al*. 2020; Mlodinow *et al*. 2022). Our results also confirmed that Asian Woollynecks are composed of 2 genetically distinct clades corresponding to the currently recognized subspecies classification, *C. e. neglecta* and *C. e. episcopus*. These 2 groups were genetically different in all the analyses: they formed 2 distinct clusters in the phylogenetic tree, grouped independently of each other in the principal component analyses, showed high genetic divergence, and were recognized as different groups in the admixture analysis. This evidence, in addition to their known morphologic differences and distribution (Mlodinow *et al*. 2022), suggests *C. e. neglecta* and *C. e. episcopus* have accumulated enough differences to be recognized as different species, not just as subspecies. This process of genetic divergence in these 2 clades of Asian Woollynecks is possibly boosted by the geographical isolation of *C. e. neglecta* in the southeast islands of Asia in association with the tectonic activity in this zone during the last 10 MY (Verstappen 1997; Hall 2009). However, comprehensive population genomics studies using additional populations from other Southeast Asian areas, such as other parts of Indonesia, Thailand, Malaysia, and Cambodia, will be necessary to evaluate whether these 2 clades on Asian Woollyneck *C. e. neglecta* should continue to be classified as subspecies or potentially regarded as distinct species. Analyzing the genetic characteristics of these populations could also offer valuable insights into the substantial ongoing decline of Woolly-necked storks in Southeast Asia (Ghimire *et al*. 2021).

The estimated time of divergence between the Asian Woollyneck *C. episcopus* and the African Woollyneck *C. microscelis* was ∼3.34 MYA, dating back to the late Pliocene era. These findings align with the divergence timings observed in other vertebrates, such as Hyaenidae, between intercontinental species in Asia and Africa during the late Pliocene era (Werdelin and Lewis 2000). Additionally, the divergence between the 2 Asian subspecies, *C. e. episcopus* from mainland Asia and *C. e. neglecta* from Indonesia, was estimated to have occurred ∼0.96 MYA during the mid-Pleistocene transition. This period, spanning from ∼1.2 to 0.8 MYA, is recognized for significant global climate shifts, including glacial cooling and sea level variations (Matsuzaki *et al*. 2023). The environmental changes associated with this transition, such as lower sea levels that exposed the Indonesian landmass (Elderfield *et al*. 2012), facilitated biotic exchange between Indonesia and mainland Asia (Vos *et al*. 1994), which coincided with the first arrival of hominins in Indonesia (Matsu’ura *et al*. 2020). The divergence of 2 Asian subspecies may be the result of allopatry caused by complex climatic events that led to the connection of Indonesia and mainland Asia during this mid-Pleistocene transition.

African Woollyneck populations demonstrated lower genetic diversity in comparison to Asian Woollynecks, as evidenced by a low genome-wide average nucleotide diversity, shorter branches in the phylogeny, and an absence of distinct structure in admixture analysis. These outcomes may suggest that the effective population size of African Woollyneck was smaller during the period when these museum samples were collected, around the 1900s. Alternatively, these results could imply ongoing gene flow among African Woollyneck populations after a recent period of divergence. Due to the lack of reliable data on population size changes in African Woollyneck over the past century and the limited availability of robust population genomics data, we are unable to provide a more specific explanation. However, anecdotal evidence indicates that African Woollynecks were regarded as endangered in South Africa until the 1990s because of the reduced population size (Clancey 1964; Cyrus and Robson 1980), which is consistent with our results.

While we did not detect evidence of population structure among African samples, our phylogenetic analysis revealed that they form 2 well-supported clades. Contrary to the expectation that populations in closer geographical proximity would exhibit greater genetic similarity, our findings indicated that individuals from Sierra Leone are closely related to those from South Africa, despite the substantial geographical distance between these 2 regions. A recent study on African Woollyneck populations has suggested that their distribution is fragmented (Gula *et al*. 2020), particularly in West Africa. The observed phylogenetic pattern in African Woollyneck could potentially be attributed to this fragmented distribution.

We found no evidence of ongoing gene flow among populations/species in this study. Although Woolly-necked storks can fly long distances, it is possible that they do not fly extensively across open water, suggesting water bodies may have played a significant role in the divergence of Asian and African species and likely divergence between *C. e. neglecta* and *C. e. episcopus*. The lack of ongoing gene flow may have contributed to reduced genetic variability and decreased population size, for example, in the Philippines, where the Asian Woollyneck seems to have been locally extinct from Luzon and neighboring islands (Ghimire *et al*. 2021). Demographic modeling using fastsimcoal provided evidence of early gene flow, but no ongoing gene flow, between Asian and African Woollynecks, as the 2 species were diverging from their common ancestor. As species begin to diverge, there may still be opportunities for genetic exchange through various mechanisms, such as hybridization, introgression, or incomplete reproductive isolation (Abbott *et al*. 2013).

Multiple lines of evidence suggest that both Asian and African Woollynecks are responding to changes in local environments and potentially undergoing local adaptation. Firstly, they demonstrate variations in response to climatic variables, including bioclimatic factors like precipitation and temperature (Gula *et al*. 2020). For instance, the probability of the presence of Asian Woollynecks increases with precipitation in the warmest quarter, whereas the trend is the opposite for African Woollynecks. The same study predicts a positive association of African Woollynecks with flooded forest/shrubland and grassland with woody vegetation, while Asian Woollynecks are associated with natural vegetation patches and croplands. Secondly, there is evidence indicating that both species are utilizing urban environments. African Woollynecks have been observed using supplementary feeding in urban areas of South Africa (Thabethe and Downs 2018), and Asian Woollynecks have been documented utilizing artificial structures for nesting in recent years (Vaghela *et al*. 2015; Hasan and Ghimire 2020). However, African Woollynecks seem to fare better in urban environments than Asian Woollynecks. Given that our samples date back ∼70 years and our sample size is limited, our ability to draw definitive conclusions about local adaptation is constrained. Nevertheless, we have identified numerous genomic regions showing strong genetic divergence between Asian and African Woollynecks that may potentially harbor genes involved in local adaptation.

This study is anticipated to create avenues for future genomic research that will use a larger sample size from populations across the distribution of Asian and African Woollynecks and examine genomic signatures of local adaptation associated with changes in their morphology, the timing of reproduction, and urbanization. We are also suggesting the need to study the diversity of Asian Woollyneck populations to determine if *C. e. neglecta* and *C. e. episcopus* are different species, and therefore, they require targeted conservation strategies to mitigate the decline and promote the recovery of Woolly-necked storks in Southeast Asia. While this study presents evidence of genomic differentiation between Asian and African Woollynecks, the specific evolutionary processes that led to their divergence and the molecular mechanisms underlying their speciation remain topics for future investigation. Studies on patterns of genetic differentiation, phylogenetics, and ecological adaptations in closely related species across continents will allow us to better understand how geological events, geographical barriers, and environmental differences shape biodiversity. Woolly-necked storks are a suitable system to explore these evolutionary dynamics.

## Supplementary Material

jkae081_Supplementary_Data

## Data Availability

All short-read whole-genome sequencing data have been submitted to the National Center for Biotechnology Information (NCBI) under BioProject accession number PRJNA996906. Scripts and workflow for all analyses done in this paper will be available via a dedicated GitHub page—https://github.com/sangeet2019/Woollyneck-storks. Supplemental material available at G3 online.
