# Supplementary material for: Museum genomics approach to study the taxonomy and evolution of Woolly-necked storks using historic specimens
Source: G3 (Bethesda). 2024 Apr 16;14(7):jkae081. doi: 10.1093/g3journal/jkae081 (PMC11771223; doi:10.1093/g3journal/jkae081)
Supplement: jkae081_Supplementary_Data [file jkae081_supplementary_data.zip › Supplemental_Figures_and_Tables_G3-2024-404829.docx]

**Supplementary Figures**

**
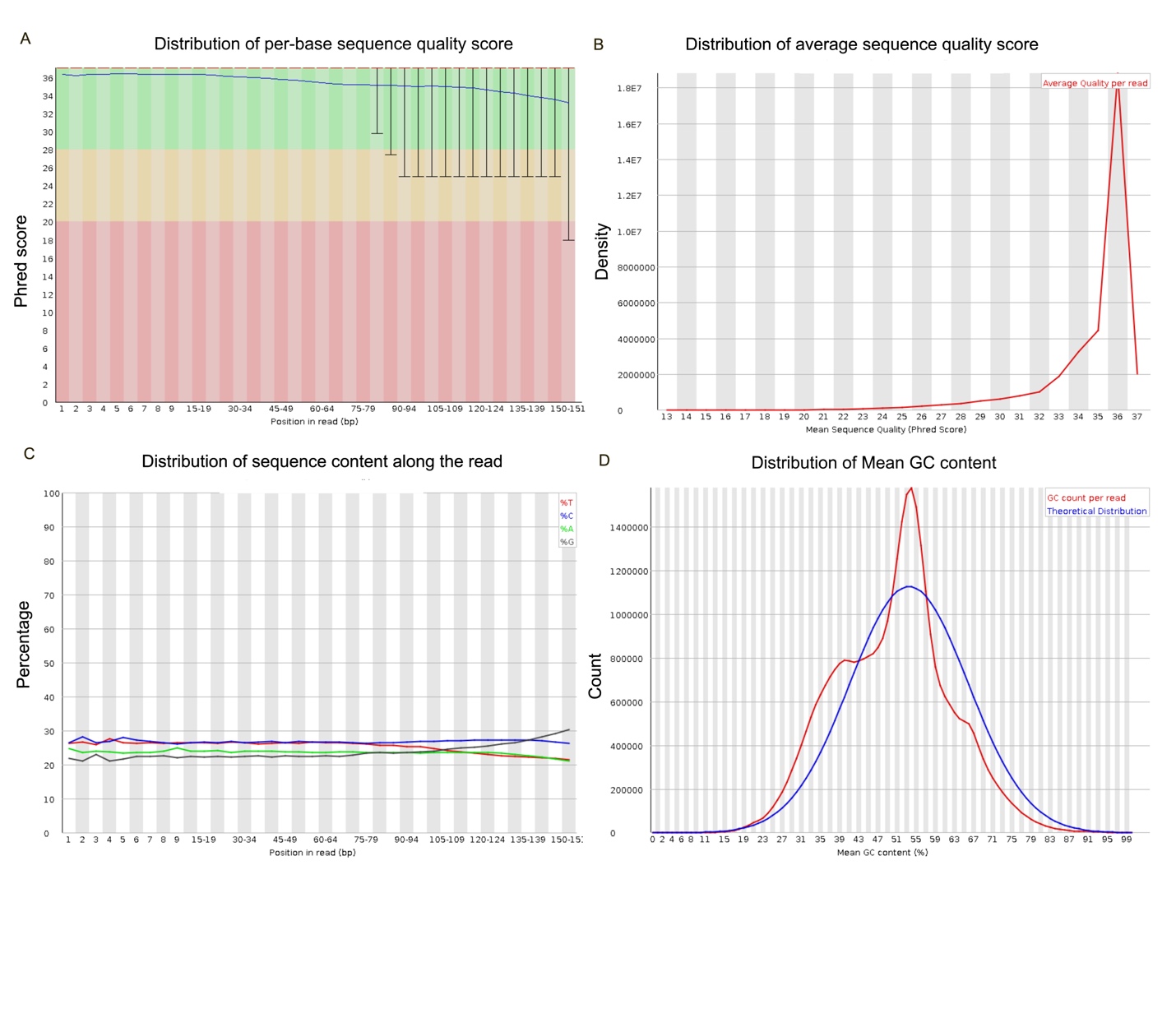
 Figure S1:** FASTQC report from sample MCZ:264670 (*Ciconia microscelis*) (A) Distribution of per-base sequence quality score (B) Distribution of average sequence quality score (C) Distribution of sequence content along the read (D) Distribution of Mean GC content. The quality report was similar for all 12 samples.


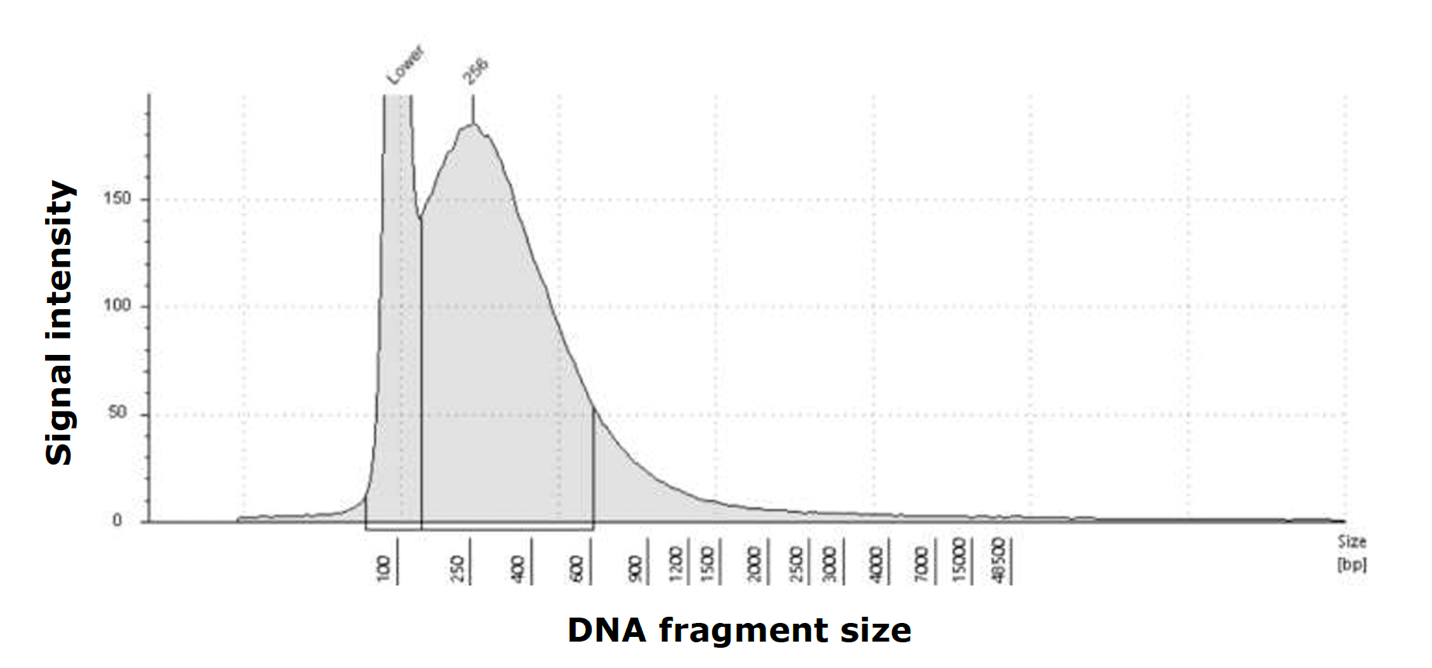


**Figure S2:** Distribution of fragment lengths of genomic DNA extracted from sample MCZ:264670 (Ciconia microscelis). Great majority of DNA had shorter fragments indicative of DNA degradation. The distribution of fragment lengths was similar for all 12 samples.


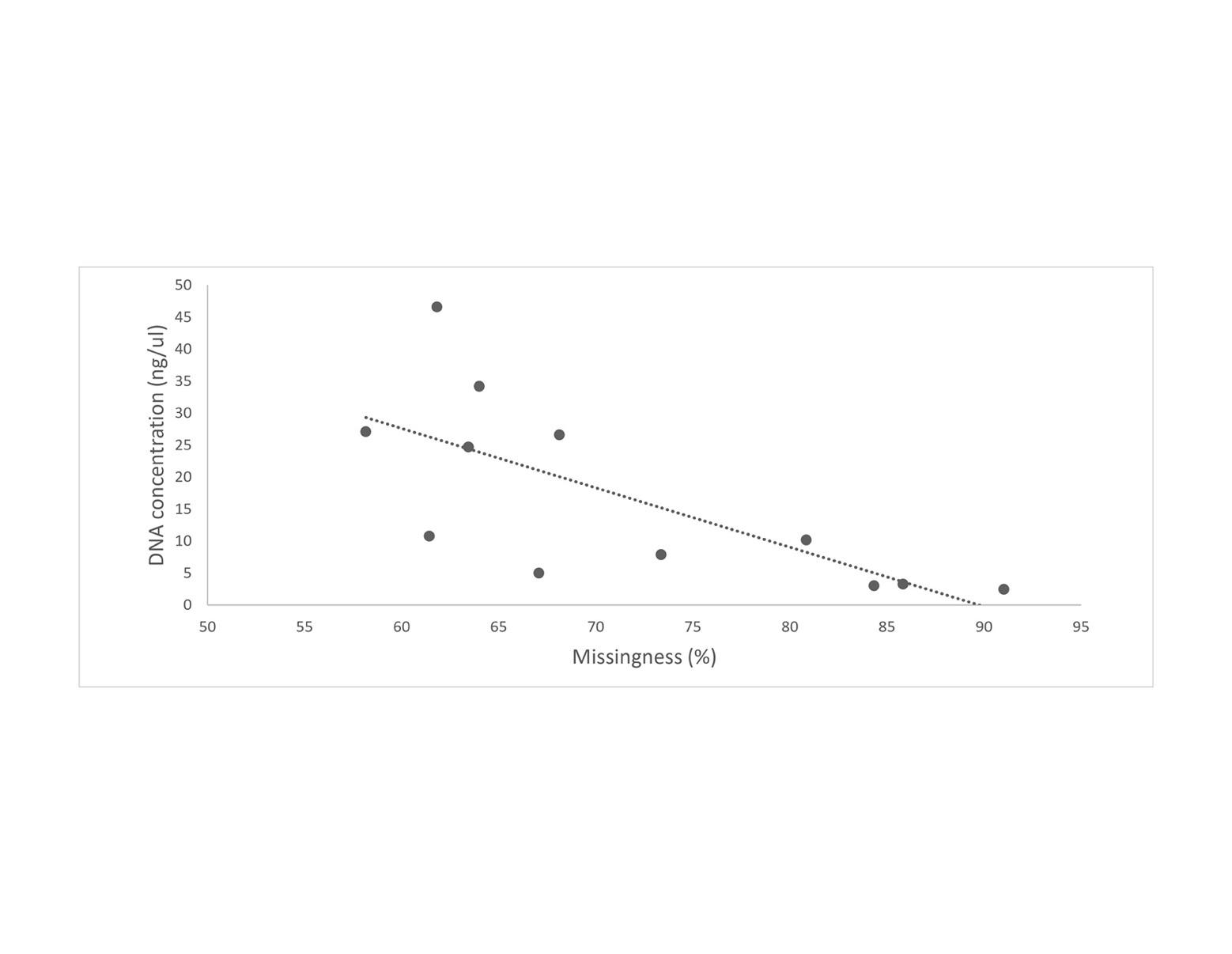


**Figure S3**: Correlation between DNA quality (measured as DNA concentration) and percentage of missing SNPs across all samples. Lower quality DNA had higher rate of missing SNPs.


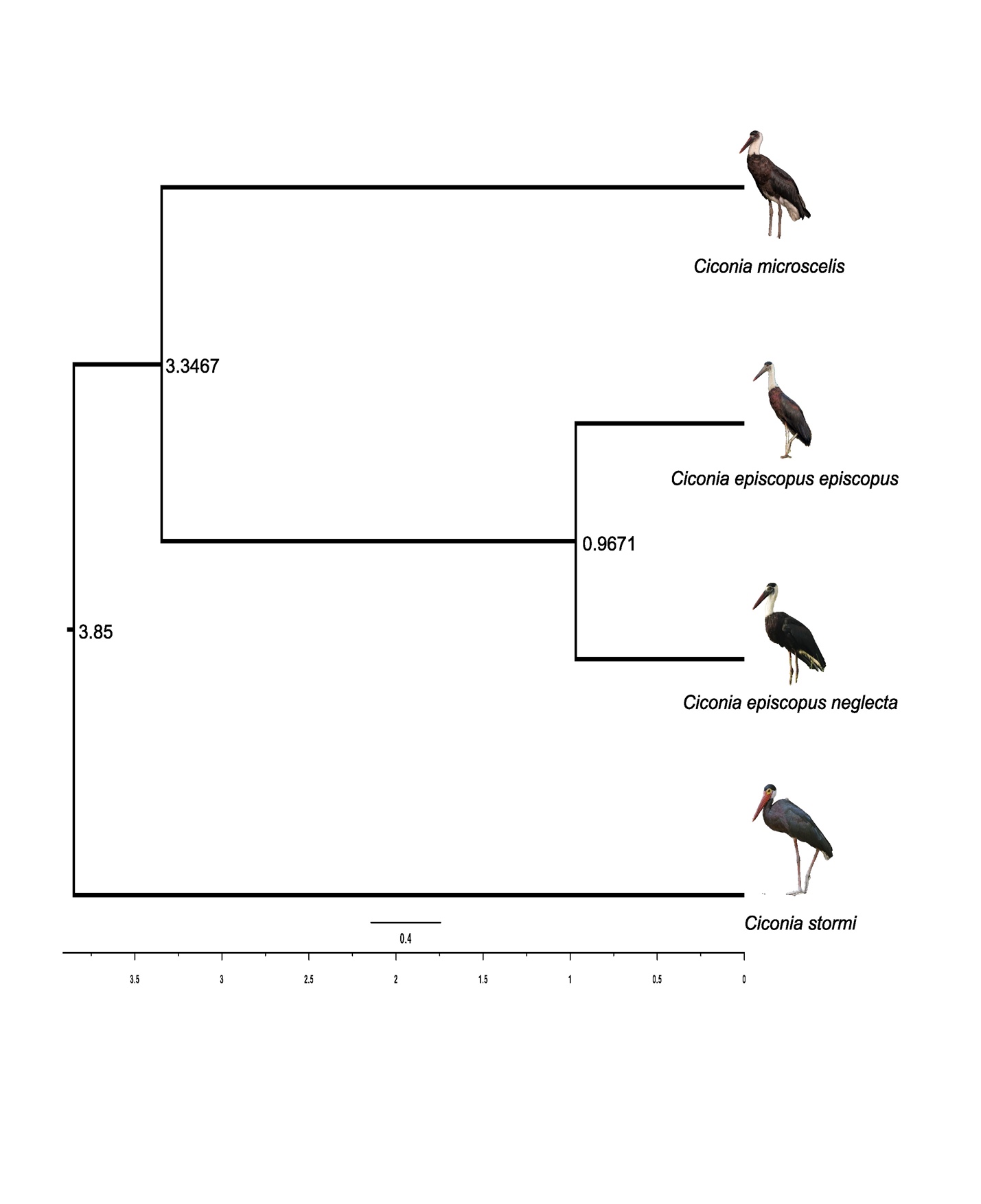


**Figure S4**: Phylogenetic tree showing divergence time estimates. Values on each node represents time since divergence million years ago (MYA).


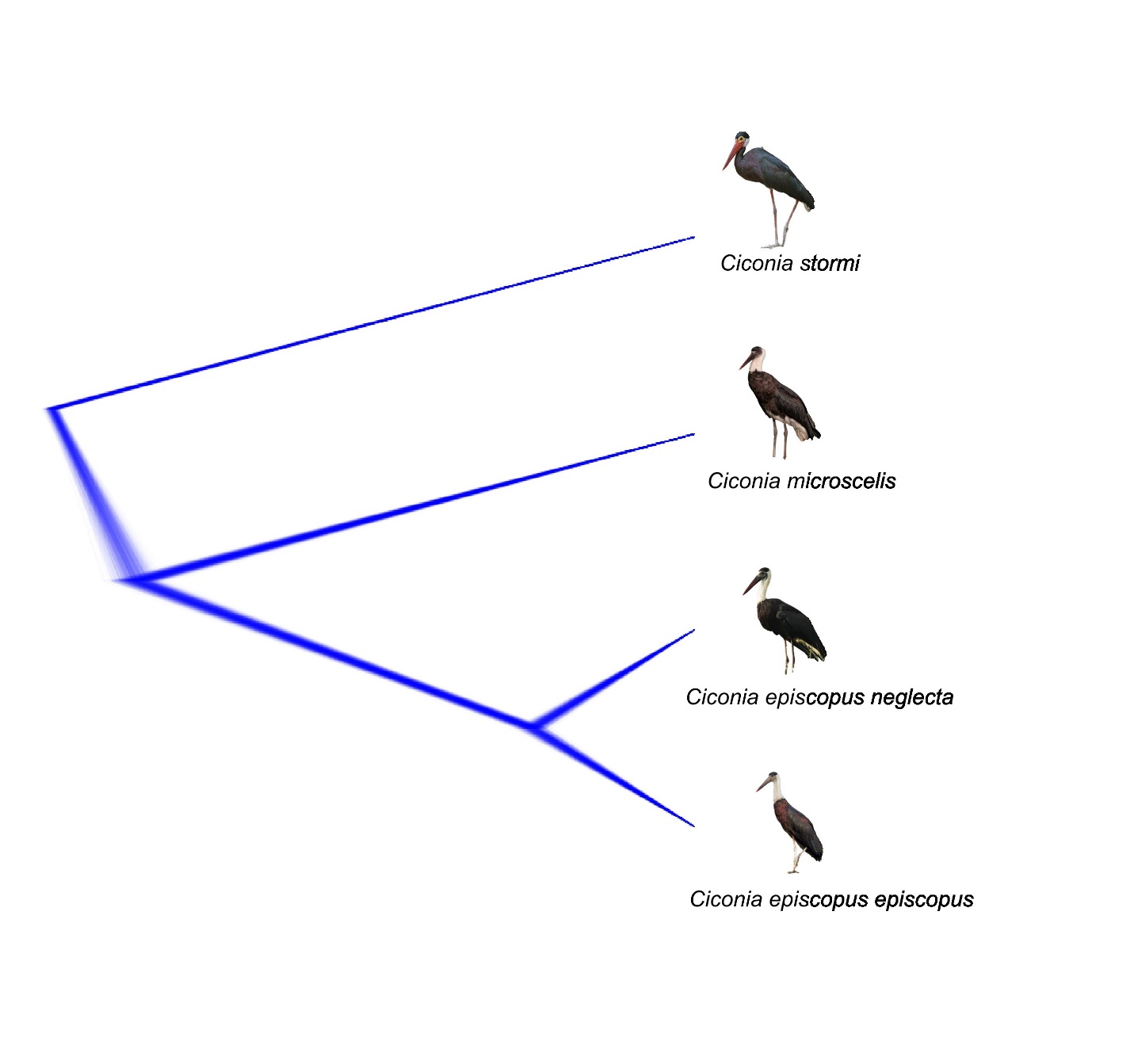


**Figure S5**: Cladogram of the posterior distribution of all “gene” trees generated using SNAPP (Bryant et al, 2012). SNAPP employs Bayesian inference to estimate the genealogy or gene tree for each locus in the dataset.


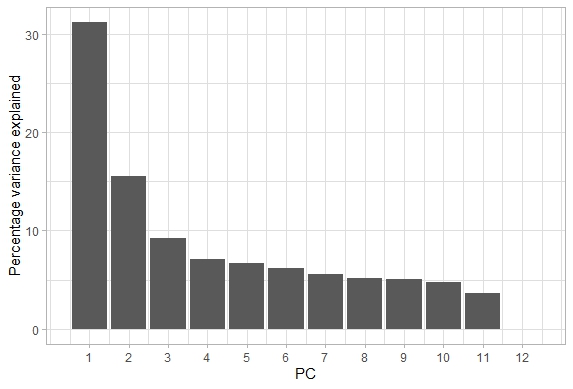


**Figure S6:** Percentage of variable explained by each 12 principal components (PCs). First two PCs explained almost 50% of genetic variations among samples.


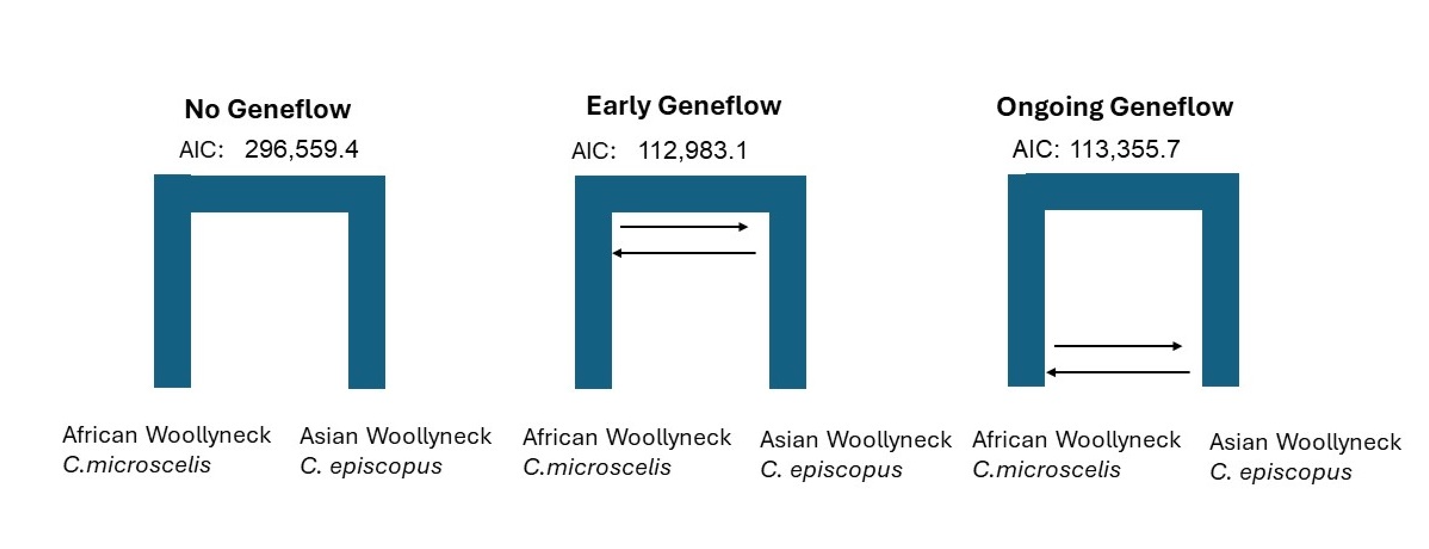


**Figure S*7*:** Possible gene flow scenarios among the Asian and African Woollyneck populations. Lowest AIC value was identified from “Early GeneFlow” supporting it as the best fit model.

**Table S1:** Details of museum specimen used in this study.

| **Species** | **MCZ^*^ Catalogue Number** | **Sampling location** | **Sampling Date** |
| --- | --- | --- | --- |
| *Ciconia microscelis* | 11616 | South Africa | NA^**^ |
| *Ciconia microscelis* | 12831 | African Origin | NA^**^ |
| *Ciconia microscelis* | 264670 | Ganta via Monrovia, Liberia | 1939 |
| *Ciconia microscelis* | 270607 | Miranja,Tanzania | 1939 |
| *Ciconia microscelis* | 279738 | Nongoba Bullom Chiefdom, Sierra Leone | 1953 |
| *Ciconia stormi* | 170531 | West Sumatra, Indonesia | 1934 |
| *Ciconia stormi* | 170532 | West Sumatra, Indonesia | 1934 |
| *Ciconia episcopus neglecta* | 270046 | Sulawesi Island, Indonesia | 1939 |
| *Ciconia episcopus neglecta* | 270047 | Sulawesi Island, Indonesia | 1939 |
| *Ciconia episcopus neglecta* | 270321 | Sulawesi Island, Indonesia | 1938 |
| *Ciconia episcopus episcopus* | 278124 | Dang District,India | 1948 |
| *Ciconia episcopus episcopus* | 54164 | Unknown, Asian Origin | 1953 |

*Museum of Comparative Zoology, Harvard University

**Not available

**Table S2:** Total amount of DNA extracted from toe-pad skins samples of historic museum specimens used in this study.

| **Species** | **MCZ^*^ Catalogue Number** | **Total amount of DNA extracted (ng)** | **Total number of sequencing reads** | **Total genomic data (GB)** |
| --- | --- | --- | --- | --- |
| *Ciconia microscelis* | 11616 | 276 | 88,042,788 | 13.21 |
| *Ciconia microscelis* | 12831 | 294 | 65,064,226 | 9.76 |
| *Ciconia microscelis* | 264670 | 321.9 | 89,185,542 | 13.38 |
| *Ciconia microscelis* | 270607 | 429 | 77,001,948 | 11.55 |
| *Ciconia microscelis* | 279738 | 156 | 87,657,360 | 13.15 |
| *Ciconia stormi* | 170531 | 210 | 69,981,572 | 10.50 |
| *Ciconia stormi* | 170532 | 124 | 73,581,904 | 11.04 |
| *Ciconia episcopus neglecta* | 270046 | 126.3 | 77,323,416 | 11.60 |
| *Ciconia episcopus neglecta* | 270047 | 162 | 86,747,950 | 13.01 |
| *Ciconia episcopus neglecta* | 270321 | 210 | 81,744,096 | 12.26 |
| *Ciconia episcopus episcopus* | 278124 | 225 | 78,198,934 | 11.73 |
| *Ciconia episcopus episcopus* | 54164 | 327 | 84,796,218 | 12.72 |

**Table S3**: Sequence alignment statistics by mapping short reads against the reference genome of Maguari stork (Ciconia maguari)

| **Species** | **MCZ^*^ Catalogue Number** | **Total sequence mapped to reference genome (%)** | **Both pair mapped to reference genome (%)** | **Average genome wide sequencing depth** |
| --- | --- | --- | --- | --- |
| *Ciconia microscelis* | 11616 | 92.33 | 84.46 | 5.69 |
| *Ciconia microscelis* | 12831 | 90.51 | 81.97 | 5.24 |
| *Ciconia microscelis* | 264670 | 98.91 | 89.96 | 6.63 |
| *Ciconia microscelis* | 270607 | 98.76 | 88.63 | 6.21 |
| *Ciconia microscelis* | 279738 | 99.12 | 85.55 | 6.16 |
| *Ciconia stormi* | 170531 | 83.2 | 76.68 | 4.5 |
| *Ciconia stormi* | 170532 | 97.54 | 91.3 | 5.59 |
| *Ciconia episcopus neglecta* | 270046 | 98.8 | 86.52 | 5.51 |
| *Ciconia episcopus neglecta* | 270047 | 98.62 | 90.25 | 6.86 |
| *Ciconia episcopus neglecta* | 270321 | 96.49 | 87.22 | 6.46 |
| *Ciconia episcopus episcopus* | 278124 | 99.34 | 87.59 | 6.23 |
| *Ciconia episcopus episcopus* | 54164 | 93.25 | 84.77 | 5.92 |

**Table S4:** Statistics of missing SNPs (Missingness score = 0, SNP genotyped across all 12 samples; and score = 1, SNP missing in all samples)

| **Missingness Score** | **Total number of SNPs** | **% of total SNPs** |
| --- | --- | --- |
| < 0.1 | 131,965 | 0.97 |
| 0.1-0.2 | 224,543 | 1.66 |
| 0.2-0.3 | 439,916 | 3.25 |
| 0.3-0.4 | 770,401 | 5.69 |
| 0.4-0.5 | 1,216,984 | 8.98 |
| 0.5-0.6 | 4,129,831 | 30.48 |
| >0.7 | 6,636,116 | 48.98 |

**Table S5:** Cross-validation error (CV) for k = 1–5 ancestry (K) shows that k= 3 or 4 are the optimal number of genetic clusters for admixture analysis.

| **Ancestry(k)** | **Cross-validation error (CVV)** |
| --- | --- |
| 1 | 0.51043 |
| 2 | 0.65317 |
| 3 | 0.53515 |
| 4 | 0.53604 |
| 5 | 0.60235 |

**Table S6:** Estimates of ABBA-BABA analysis using Dsuite (Malinsky et al. 2021) to identify evidence of gene flow between Asian Wollynecks (C. neglecta and C. episcopus) and African Woollyneck (C. microcelis) using Storm’s stork (C. stormi) as an outgroup.

| **P1** | **P2** | **P3** | **D statistics** | **Z-score** | **p-value** | **f4-ratio** | **BBAA** | **ABBA** | **BABA** |
| --- | --- | --- | --- | --- | --- | --- | --- | --- | --- |
| *Ciconia episcopus neglecta* | *Ciconia episcopus episcopus* | *Ciconia microscelis* | 0.014 | 1.42 | 0.07 | 0.029 | 1156.02 | 648.246 | 629.854 |
